# Confirmatory Efficacy and Safety Trial of Magnetic Seizure Therapy for Depression (CREST-MST): protocol for identification of novel biomarkers via neurophysiology

**DOI:** 10.1186/s13063-021-05873-7

**Published:** 2021-12-11

**Authors:** Zafiris J. Daskalakis, Shawn M. McClintock, Itay Hadas, Elisa Kallioniemi, Reza Zomorrodi, Alanah Throop, Lucy Palmer, Faranak Farzan, Kevin E. Thorpe, Carol Tamminga, Daniel M. Blumberger

**Affiliations:** 1 Department of Psychiatry, University of California, San Diego, La Jolla, CA USA; 2 Department of Psychiatry, University of Texas Southwestern Medical Center, Dallas, TX USA; 3 Temerty Centre for Therapeutic Brain Intervention, Centre for Addiction and Mental Health, Toronto, Ontario Canada; 4 School of Mechatronic Systems Engineering, Simon Fraser University, Surrey, British Columbia Canada; 5 Applied Health Research Centre, Li Ka Shing Knowledge Institute of St. Michael’s, Toronto, Ontario Canada; 6 Dalla Lana School of Public Health, University of Toronto, Toronto, Ontario Canada; 7 Institute of Medical Science and Department of Psychiatry, University of Toronto, Toronto, Ontario Canada

**Keywords:** Treatment-resistant depression, Magnetic seizure therapy, Electroconvulsive therapy, Transcranial magnetic stimulation, Electroencephalography, Cortical inhibition, Neurophysiology

## Abstract

**Background:**

Electroconvulsive therapy (ECT) is the most effective treatment for treatment-resistant depression (TRD), especially for acute suicidal ideation, but the associated cognitive adverse effects and negative stigma limit its use. Another seizure therapy under development is magnetic seizure therapy (MST), which could potentially overcome the restrictions associated with ECT with similar efficacy. The neurophysiological targets and mechanisms of seizure therapy, however, remain poorly understood.

**Methods/design:**

This neurophysiological study protocol is published as a companion to the overall Confirmatory Efficacy and Safety Trial of Magnetic Seizure Therapy for Depression (CREST-MST) protocol that describes our two-site, double-blind, randomized, non-inferiority clinical trial to develop MST as an effective and safe treatment for TRD. Our aim for the neurophysiological component of the study is to evaluate two biomarkers, one to predict remission of suicidal ideation (primary outcome) and the other to predict cognitive impairment (secondary outcome). Suicidal ideation will be assessed through cortical inhibition, which according to our preliminary studies, correlates with remission of suicidal ideation. Cortical inhibition will be measured with simultaneous transcranial magnetic stimulation (TMS) and electroencephalography (EEG), TMS-EEG, which measures TMS-evoked EEG activity. Cognitive adverse effects associated with seizure therapy, on the contrary, will be evaluated via multiscale entropy analysis reflecting the complexity of ongoing resting-state EEG activity.

**Discussion:**

ECT and MST are known to influence cortical inhibition associated with depression, suicidal ideation severity, and clinical outcome. Therefore, evaluating cortical inhibition and brain temporal dynamics will help understand the pathophysiology of depression and suicidal ideation and define new biological targets that could aid clinicians in diagnosing and selecting treatments. Resting-state EEG complexity was previously associated with the degree of cognitive side effects after a seizure therapy. This neurophysiological metric may help clinicians assess the risk for adverse effects caused by these useful and effective treatments.

**Trial registration:**

ClinicalTrials.govNCT03191058. Registered on June 19, 2017.

## Background

The need to optimize our therapeutic approach and provide optimal treatment options for depression is critical. While electroconvulsive therapy (ECT) is a well-established and highly effective treatment with remission rates that range from 60 to 80% [1], less than 1% of adult patients with treatment-resistant depression (TRD) receive ECT in the USA and Canada [2, 3]. One of the major reasons that providers, patients, and their families refuse to consider ECT—even when confronted with a chronic and disabling depression that may be unresponsive to other treatments—is the concern about associated cognitive side effects. Potential cognitive impairment includes anterograde and retrograde amnesia and prolonged post-treatment disorientation that is distressing to patients and mitigates antidepressant benefits [4–8].

A viable antidepressant seizure therapy under development is magnetic seizure therapy (MST), which relative to ECT has comparable response/remission rates and a more favorable cognitive side effect profile than ECT [9–11]. MST is based on the principles of ECT and repetitive transcranial magnetic stimulation (rTMS) with the aim of producing a therapeutic seizure with rapidly alternating magnetic fields. Magnetic fields deliver the seizure-inducing electric field directly to the cortex and not on the scalp similar to ECT, allowing more focal and controlled targeting of the electric field [12, 13]. Furthermore, magnetic fields only reach the outermost layers of the cortex and do not spread to subcortical structures, which may play a role in the cognitive safety profile of MST [14].

Despite the exact seizure induction method, the biological targets of seizure therapy remain mostly unknown, which limits the development of individualized treatments. Indeed, finding reliable biological targets is essential for individualized care. For the treatment of TRD and associated suicidal ideation, evidence suggests that prefrontal γ-aminobutyric acid (GABA)ergic inhibitory neurotransmission as measured by cortical inhibition may represent an important neurophysiological target [15]. An understanding of the mechanisms of seizure therapy would allow for the identification of patients most likely to respond to the treatment and provide tools to develop alternative treatments for non-responders. Neurophysiological exploration in this patient population will also serve to deepen and enrich our biological models of depression.

This paper presents the neurophysiological biomarker protocol embedded within the National Institute of Mental Health (NIMH) Confirmatory Efficacy and Safety Trial of Magnetic Seizure Therapy for Depression (CREST-MST) randomized, double-blind, non-inferiority trial [16], designed to compare the efficacy of MST with ECT in patients with TRD. The full CREST-MST study protocol (research protocol version 12.0; 21-Oct-2020) can be found in an accompanying publication, with further details on the non-inferiority trial design and outcomes [16]. In addition to treatment efficacy, the CREST-MST study evaluates suicidal ideation—a symptom domain in TRD that has tremendous public health impact—as an outcome variable of interest. In the neurophysiological component of this study, we will evaluate cortical inhibition as a potential neurophysiological mechanism through which MST and ECT may result in attenuation of suicidal ideation. Furthermore, to gain understanding of the seizure therapy associated cognitive adverse effects, as a secondary outcome, we will evaluate the potential of a neurophysiological biomarker reflecting the complexity of brain temporal dynamics in predicting cognitive adverse effects.

## Methods/design

### Study design and setting

The purpose of this study protocol is to outline the neurophysiological methods utilized to address the specific aims related to biomarkers as part of CREST-MST [16]. The CREST-MST trial is registered with ClinicalTrials.gov under the identifier NCT03191058 and results will be reported in a manner consistent with the international Consolidated Standards of Reporting Trials (CONSORT) guidelines. This protocol is in accordance with the Standard Protocol Items: Recommendations for Interventional Trials (SPIRIT) guidelines [17] (see CREST-MST Protocol [16]). Seizure therapies and human neurophysiological recordings are conducted at two leading academic institutions in North America: the Temerty Centre for Therapeutic Brain Intervention at the Centre for Addiction and Mental Health in Toronto, Ontario, Canada, and University of Texas Southwestern Medical Center in Dallas, TX, USA.

### Recruitment and retention

All patients enrolling in CREST-MST are recruited to participate in the optional neurophysiology component of the trial. A brief explanation of the measurement is provided to the patient and all questions are answered by a neurophysiology expert. Patients are asked to participate in this experimental measure before the intervention begins and within 4 days of the final treatment. All patients terminating the study, whether they are completed or exiting prematurely, are automatically scheduled for their post-treatment neurophysiology appointment.

### Eligibility criteria

Individuals who meet the eligibility criteria for the CREST-MST study [16] are given the option to participate in the neurophysiology component of the trial. Consenting to this part of the study is optional and does not affect a participant’s status in CREST-MST. Specifically, the rights and welfare of the participants are protected by emphasizing to them that the quality of their medical care and that their participation in the CREST-MST study will not be adversely affected if they decline to participate in this aspect of the trial.

### Informed consent procedures

The CREST-MST consent form describes in detail the additional neurophysiological testing sessions and any risks associated with this component of the study. A verbal explanation is also provided in terms suited to the individual’s comprehension, including a brief overview of the procedures involved in the transcranial magnetic stimulation (TMS) combined with electroencephalography (EEG) (TMS-EEG) sessions. Written documentation of informed consent for this additional testing session is required. Consent for the neurophysiology measurement is obtained in accordance with the Institutional Review Board (IRB) and Good Clinical Practice/Tri-Council (GCP) guidelines within the CREST-MST consent document. Patients are informed of any approved protocol changes at their next study visit and re-consented on the updated procedures.

### Study schedule

Following the CREST-MST screening visit, and upon confirmation of full eligibility, the participant will complete clinical, cognitive, and neurophysiological outcome measures within 1 week prior to the first study ECT/MST treatment. Post-treatment measures will be collected within four days after the last study ECT/MST treatment. A detailed description of the CREST-MST study measures and schedule is outlined in the original trial [16]. Raters inquire about adverse events (AE) at every study visit and all events endorsed by a patient are recorded in the study database.

### Neurophysiological procedures

Neurophysiological measurements are conducted with trained and certified personnel familiar with all protocol requirements and safe administration of TMS. Research staff conducting the measurement are blind to treatment randomization. The neurophysiological measurements consist of three parts: (1) resting-state EEG, (2) motor “hotspot” mapping and motor threshold determination, and (3) simultaneous frontal TMS-EEG. EEG is measured with a 64-channel TMS-compatible amplifier, QuikCap EEG cap, and Curry 8 data acquisition software (all EEG parts are from Compumedics Neuroscan, Charlotte, NC, USA). Muscle activity is measured with electromyography (EMG) using two disposable surface electrodes placed in a belly-to-tendon arrangement over the right abductor pollicis brevis (APB) muscle. EMG is amplified (Intronix Technologies Corporation Model 2024F, Bolton, Ontario, Canada), filtered (band-pass 2 Hz to 5 kHz), digitized at 5 kHz (Micro 1401, Cambridge Electronic Design, Cambridge, UK), and further processed online (Signal Software version 7, Cambridge Electronic Design, UK). TMS pulses are administered with a Magstim BiStim stimulator (Magstim Company Ltd., UK) using a figure-eight coil (D70 Alpha Flat Coil, 70 mm winding) and a monophasic stimulation waveform.

First, 10 min of resting-state EEG is measured with 5 min eyes closed and 5 minutes eyes open. Resting-state EEG is recorded at 1000 Hz sampling frequency. Subsequently, the motor “hotspot,” the cortical location evoking the largest amplitude motor evoked potential (MEP) in the right APB muscle, is located. At this location, the resting motor threshold is defined as the minimum TMS intensity eliciting an MEP of at least 50 μV in five out of ten stimulations [18]. At the hotspot, the TMS intensity producing a 1-mV response as the average of ten TMS pulses is also measured. This intensity is used as the stimulation intensity in the TMS-EEG component and used to compensate for the longer coil-to-cortex distance at the frontal brain areas compared to the motor cortex. If the 1-mV intensity cannot be determined, i.e., the intensity is higher than 100% of maximum stimulator output (MSO), then 100% of MSO is set as the intensity. TMS-EEG is targeted to the left dorsolateral prefrontal cortex (DLPFC), located with the Beam F3 method [19] by keeping the coil angle approximately at 45 degrees from the vertex, and is recorded in two conditions: (1) single-pulse and (2) paired-pulse. Both conditions consist of 100 trials repeated every 5 s. The inter-pulse interval in paired-pulse is 100 ms. TMS-EEG data is sampled at 20,000 Hz, and during the measurement, auditory masking is played via earplugs (ER1 Insert Earphones, Etymotic, USA) to minimize the TMS pulse auditory sounds. The earplugs also provide auditory protection for the participant. The order of eyes closed and eyes open resting-state EEG and single- and paired-pulse TMS-EEG is randomized between participants. The same order is maintained for pre-and post-ECT/MST intervention sessions with the same participant.

### Outcomes

The objective of this study protocol is to evaluate two neurophysiological biomarkers within the context of the wider CREST-MST parent study: (1) To predict remission of suicidal ideation via TMS-EEG (primary outcome), (2) To predict treatment-induced cognitive impairment with resting-state EEG (secondary outcome). Prediction of suicidal ideation remission will be evaluated from the TMS-EEG measures reflecting cortical inhibition. Prediction of cognitive impairment will be made from resting-state EEG via multiscale entropy (MSE) analysis, which quantifies the complexity of brain temporal dynamics.

#### Primary neurophysiological outcome: predicting remission of suicidal ideation

Treatment response for depression has been associated with changes in the function of GABAergic interneurons, particularly in the left DLPFC [20]. Likewise, improvement in suicidal ideation has been related to changes in GABAergic function [20, 21]. To evaluate changes in cortical inhibition and predict remission of suicidal ideation, we will evaluate the N100 response from single-pulse TMS-EEG and the long-interval intracortical inhibition (LICI) comparing paired-pulse TMS-EEG to single-pulse. N100 is quantified as the amplitude of the negative peak between 90 and 130 ms in the TMS-evoked potential (TEP) after the TMS-pulse. LICI is quantified as the amount of suppression in the paired-pulse TEP relative to the single-pulse TEP in a time-window of 50–500 ms after the TMS-pulse.

Preliminary data from our group in adult patients with TRD (*n*=27) undergoing MST demonstrated that the amplitude of the N100 and the extent of LICI in the frontal and central midline region significantly correlate with remission of suicidal ideation based on the Beck Scale for Suicidal Ideation (SSI). This relationship was specific to the left DLPFC and was not observed when N100 and LICI were assessed in the left motor cortex. We demonstrated that N100 and LICI in the frontal cortex predicted remission of suicidal ideation with 90% sensitivity and 89% specificity [20]. In another study, we demonstrated that change in left DLPFC LICI predicted remission of suicidal ideation correctly with 85.7% sensitivity and 100% specificity (area under the receiver operating characteristic curve (ROC) = 0.98, *p* = 0.002) in a population of 20 TRD patients undergoing MST [21]. This pilot data suggests that LICI is modified by seizure therapy and that individualized changes in LICI by seizure therapy may underlie the differential treatment response for suicidal ideation.

#### Secondary neurophysiological outcome: predicting ECT-induced cognitive impairment through multiscale entropy

Seizures influence the temporal dynamics of brain activity. These dynamics reflect information processing associated with affective and cognitive processes, and thereby, these dynamics can be interpreted as indications of the brain state [22]. To quantify the complexity of these dynamics, we use a method called MSE [23] to analyze the data from a resting-state EEG measurement. The more complex the EEG signal, the fewer recurring temporal patterns it contains.

Preliminary data from our group suggested that MSE can be used to predict cognitive adverse effects following seizure therapy [24]. Resting-state EEG was obtained from adult patients with TRD (*n*=34) receiving MST or ECT, and MSE was employed before and after seizure therapy. We found that following ECT, but not MST, the complexity of brain temporal dynamics in coarse time scales was significantly increased. In a subset of 19 patients, we also obtained scores of global cognitive functions using the Montreal Cognitive Assessment (MoCA) before and after ECT (*n* = 6) and MST (*n* =13) treatment. We found that increased complexity in coarse time scales derived through MSE predicted change in the MoCA total score with over 90% accuracy. Farzan et al. [24] provide a detailed description of the MSE analysis and previous findings.

### Statistical methods

A full outline of the non-inferiority trial design and statistical analysis is included in the CREST-MST protocol manuscript [16]. Statistical methods specific to this companion biomarker study are outlined below:

Based on the variance of the cortical inhibition metrics (i.e., N100 and LICI), a linear discriminant model will be constructed to predict suicidality remission per participant (remission defined as SSI score reduction from 1 or higher before treatment to 0 after treatment). The discriminative properties of this model will be evaluated through the area under the curve (AUC) of a ROC curve analysis. ROC analysis is used in medicine to evaluate diagnostic test accuracy. The ROC involves plotting the sensitivity (true positive rate) versus 1-specificity (false positive rate). An AUC of 1 represents perfect prediction with optimal sensitivity and specificity, while an AUC of 0.5 suggests that the diagnostic test is no better than chance at diagnostic prediction. For our purposes, the ROC AUC corresponds to the change in cortical inhibition that optimally distinguishes MST and ECT remitters from non-remitters (defined as a score of 0 on the SSI). Based on preliminary data the change in cortical inhibition (defined as the LICI and N100) will be used to distinguish MST and ECT SSI remitters from non-remitters using a ROC AUC of > 0.85 on all frontal electrodes [20]. A false detection rate correction is applied to all frontal electrodes.

For evaluating the cognitive adverse effects, we will investigate the relationship between change in complexity in coarse time scales derived through MSE and change in the Autobiographical Memory Test (AMT) scores. The ROC analysis will be conducted to evaluate this biomarker's accuracy in explaining the cognitive adverse events associated with seizure therapy according to previous literature [24].

### Sample size

The total sample size of the CREST-MST trial is anticipated to be 260 participants (130 per treatment group), which was derived as a function of tolerance and power with a significance level of 0.05. Further details relating to sample size and significance can be found in the overall CREST-MST protocol [16]. The sample size for the neurophysiological analysis is based on the CREST-MST protocol to power the analysis with a comparable significance level of 0.05, yielding 80% power to detect a difference in SSI of 0.4 [16]. This is sufficient to provide evidence of an association between change in cortical inhibition and suicidal ideation.

### Quality control

To ensure high-quality EEG data, the electrode impedances are kept below 5 kΩ during the measurement. Participants are asked to avoid excessive blinking and body movement and to keep their facial muscles as relaxed as possible. The participants are also asked to focus their gaze on a black cross in front of them to avoid any unnecessary eye movement. Before the TMS-EEG, the target location is also tested. This is done by giving single-pulse TMS to the defined target location and measuring the duration of the TMS-evoked muscle decay artifact in the EEG. If this is more than 20 ms, other coil angles and/or locations are tested, and the target location is adjusted to minimize the muscle artifact duration. The amount of blinking caused by TMS is also considered while optimizing the target location. To reduce artifacts caused by electrode movement, a thin layer of foam is used between the coil and electrodes.

Collecting TMS-EEG neurophysiological recordings is a demanding task that requires high proficiency. To optimize our neurophysiological experimental yield, equalize signal properties between different trial centers, and for quality assurance purposes, we implemented a MATLAB pipeline that identifies serious artifacts in the EEG recording and corrects the faulty procedure immediately. The MATLAB quality control pipeline includes the following steps: (1) checking file naming convention, (2) checking recording file size, (3) estimating muscle decay artifact duration, (4) identifying silent channels, (5) identifying channels carrying 60 Hz noise, (6) identifying channels with extremely high amplitudes, (7) identifying bridged channels, (8) checking for the total number of obvious pulses, and (9) estimating the number of bad pulse epochs.

### Safety monitoring

We engage in proactive site and safety monitoring with established standard operating safety protocols as part of the standard CREST-MST study processes [16]. Neurophysiological recordings are not associated with any known risks, and there is no evidence of either short- or long-term side effects. However, some scalp discomfort during the procedure can occur in a small proportion of participants. Single- and paired-pulse TMS have been found to pose no significant health risk to properly screened participants [18], and they have been found safe even in infants and children [25, 26].

## Discussion

In this paper, we present the accompanying neurophysiological biomarker study protocol to the NIMH-funded CREST-MST double-blind, randomized, non-inferiority trial [16]. In the neurophysiological portion of the trial, we aim to evaluate two biomarkers including cortical inhibition and brain temporal dynamics. Our primary outcome is a TMS-EEG neurophysiological approach to examine whether cortical inhibition predicts remission of suicidal ideation from treatment. Our secondary outcome is a resting-state EEG approach to investigate whether cognitive adverse effects associated with seizure therapy treatments are linked with changes in the complexity of brain temporal dynamics to predict cognitive adverse effects.

ECT is the most effective treatment for TRD [1] and evidence suggests that suicidal ideation relative to other depressive symptoms may be more responsive to ECT [27, 28]. ECT is often used as a first-line treatment in TRD patients with significant suicidal ideation [27], a symptom construct that is of major public health concern and associated with the TRD phenotype. However, ECT-related cognitive adverse effects and stigma often prevent individuals from accessing this highly beneficial treatment. MST is a new seizure-inducing treatment with evidence suggesting comparable antidepressant efficacy to ECT [9–11]. MST has minimal cognitive side effects, a feature that makes it a potentially preferable treatment alternative for TRD and suicide risk [29, 30]. Identifying biomarkers and finding the neuronal mechanisms that drive ECT and MST treatment efficacy and the associated cognitive side effect profile could help predict clinical outcome and inform treatment selection. Additionally, such biomarkers could provide a better understanding of TRD pathophysiology.

Suicidal ideation has been linked to the cortical inhibitory circuits, and GABA aberrant expression is repeatedly associated with depression and suicidality [15, 31–35]. For example, Merali et al. [33] found that expression of the GABA_A_ was reduced in depressed suicide victims compared to controls. Poulter et al. [34] reported that GABA_A_ receptors were under-expressed in individuals with major depressive disorder who completed suicide compared to individuals who did not die as a result of suicide. Also, relative to non-depressed suicide individuals, the expression of several genes related to the GABA_A_ receptor is increased in depressed suicide individuals [35]. TMS can provide an index of GABA receptor-mediated inhibition in the cortex because it differentially activates inhibitory interneurons and pyramidal neurons [36]. These different activities can be measured separately by EEG. TMS-EEG can reliably evaluate cortical excitability and inhibition-related metrics (i.e., TEP-N100 and LICI), and these metrics have repeatedly been associated with TRD severity, suicidality resolution, and neuromodulatory treatment responsivity to ECT and MST [20, 21, 24, 37]. For instance, relative to non-TRD, TRD was associated with greater deficits in cortical inhibition mediated by GABA_A_ inhibitory interneurons, as measured by TMS-EEG GABAergic associated LICI and TEP-N100 [15, 20, 21]. Therefore, for the treatment of suicidal ideation, prefrontal GABAergic inhibitory neurotransmission evaluated with TMS-EEG represents an important, non-invasive, and clinically useful neurophysiological target, which will help inform our understanding of treatment response as part of the CREST-MST study.

In addition to cortical inhibition, resting-state EEG will be examined as a new biological target of seizure therapy using MSE. Data from our group suggests depressive symptoms and cognitive adverse effects following a course of seizure therapy could be predicted by evaluating the complexity of brain temporal dynamics [24]. Temporal complexity represents the temporal fluctuations in resting-state neurophysiological signals. In ECT, but not MST, we found that the complexity of brain temporal dynamics in coarse time scales was significantly increased. Greater global increase in complexity of coarse time scales for ECT patients was linked with greater decline in cognition suggesting that seizure therapy indeed modulates brain temporal dynamics [24]. Resting-state EEG will therefore allow us to assess the brain temporal dynamics and cognitive outcomes between ECT and MST in a blinded approach.

Besides the primary and secondary study outcomes, the neurophysiological data will provide additional approaches to understanding seizure therapy and TRD pathophysiology. Previously, resting-state EEG has been used for assessing functional connectivity associated with various TRD treatments and their clinical efficacy [24, 36, 38]. TMS-EEG has also been used to evaluate activation and effective connectivity at deeper brain regions associated with TRD symptoms, treatment efficacy, and treatment side effects [38, 39]. In exploratory analyses, we will utilize these approaches with our data to evaluate potential valuable TRD biomarkers.

In summary, this proposal will have a transformative, real-world clinical impact if MST, relative to ECT, demonstrates non-inferior efficacy in conjunction with a superior cognitive safety profile [16]. Biomarkers of convulsive therapy response could further improve treatment efficacy by identifying the patients who are most likely to benefit from these treatments and those at risk for adverse cognitive sequelae. In this regard, we will evaluate a promising neurophysiological biomarker—cortical inhibition—that has previously been able to predict treatment response through remission of suicidal ideation following a course of seizure therapy. Additionally, we will evaluate the potential of using the complexity of brain temporal dynamics to understand the mechanisms of cognitive adverse effects. Thus, successful completion of this study protocol may provide two new neurophysiological biomarkers for evidenced-based and precision medicine approaches in psychiatry.

## Trial status

Enrollment for this study began on June 26, 2018, and is estimated to complete recruitment by July 2024. At the time of submission, we have enrolled and randomized 117 participants. Registration: June 19, 2017 (NCT03191058), https://clinicaltrials.gov/ct2/show/NCT03191058.

## Data Availability

The final dataset generated from the current protocol will be available from the corresponding author on reasonable request.

## Abbreviations

AMT: Autobiographical Memory Test
APB: Abductor pollicis brevis
CONSORT: Consolidated Standards of Reporting Trials
CREST-MST: Confirmatory Efficacy and Safety Trial of Magnetic Seizure Therapy for Depression
DLPFC: Dorsolateral prefrontal cortex
ECT: Electroconvulsive therapy
EEG: Electroencephalography
EMG: Electromyography
GABA: γ-Aminobutyric acid
GCP: Good Clinical Practice
IRB: Institutional Review Board
LICI: Long-interval intracortical inhibition
MEP: Motor evoked potential
MST: Magnetic seizure therapy
MoCA: Montreal Cognitive Assessment
MSO: Maximum stimulator output
MSE: Multiscale entropy
NIMH: National Institute of Mental Health
ROC: Receiver operating characteristic
ROC AUC: Area under ROC curve
rTMS: Repetitive transcranial magnetic stimulation
SSI: Beck Scale for Suicidal Ideation
SPIRIT: Standard Protocol Items: Recommendations for Interventional Trials
TMS: Transcranial magnetic stimulation
TMS-EEG: Transcranial magnetic stimulation and electroencephalography
TEP: TMS-evoked potential
TRD: Treatment-resistant depression

## References

[1] Nobler, M.S. and H.A. Sackeim, Electroconvulsive therapy. Contemporary Psychiatry, ed. F.A. Henn, et al. 2001, Berlin: Springer.

[2] Hermann RC (1995). Variation in ECT use in the United States. Am J Psychiatr.

[3] Rapoport MJ, Mamdani M, Herrmann N (2006). Electroconvulsive therapy in older adults: 13-year trends. Can J Psychiatry.

[4] Sackeim HA (2008). Effects of pulse width and electrode placement on the efficacy and cognitive effects of electroconvulsive therapy. Brain Stimul.

[5] Leechuy I, Abrams R, Kohlhaas J (1988). ECT-induced postictal delirium and electrode placement. Am J Psychiatry.

[6] Sobin C (1995). Predictors of retrograde amnesia following ECT. Am J Psychiatry.

[7] Steif BL (1986). Effects of depression and ECT on anterograde memory. Biol Psychiatry.

[8] U.S..Department of Health and Human Services, Electroconvulsive Therapy (ECT) Devices for Class II Intended Uses, Food and Drug Administration, 2015: https://www.federalregister.gov/documents/2015/12/29/2015-32592/neurological-devices-reclassification-of-electroconvulsive-therapy-devices-intended-for-use-in.

[9] McClintock SM (2011). A systematic review of the neurocognitive effects of magnetic seizure therapy. Int Rev Psychiatr.

[10] Fitzgerald PB (2013). Pilot study of the clinical and cognitive effects of high-frequency magnetic seizure therapy in major depressive disorder. Depress Anxiety.

[11] Kayser S (2011). Antidepressant effects, of magnetic seizure therapy and electroconvulsive therapy, in treatment-resistant depression. J Psychiatr Res.

[12] Davey K (2003). Modeling the effects of electrical conductivity of the head on the induced electric field in the brain during magnetic stimulation. Clin Neurophysiol.

[13] Epstein CM (1990). Localizing the site of magnetic brain stimulation in humans. Neurology.

[14] McClintock SM (2013). Disruption of component processes of spatial working memory by electroconvulsive shock but not magnetic seizure therapy. Int J Neuropsychopharmacol.

[15] Levinson AJ (2010). Evidence of cortical inhibitory deficits in major depressive disorder. Biol Psychiatry.

[16] Daskalakis, ZJ, et al. Confirmatory Efficacy and Safety Trial of Magnetic Seizure Therapy for Depression (CREST-MST): study protocol for a randomized non-inferiority trial of magnetic seizure therapy versus electroconvulsive therapy. Trials 22, 786 (2021). 10.1186/s13063-021-05730-7. 10.1186/s13063-021-05730-7 PMC8576983 34749782

[17] Chan AW (2013). SPIRIT 2013 explanation and elaboration: guidance for protocols of clinical trials. BMJ.

[18] Rossini PM (2015). Non-invasive electrical and magnetic stimulation of the brain, spinal cord, roots and peripheral nerves: Basic principles and procedures for routine clinical and research application. An updated report from an I.F.C.N. Committee. Clin Neurophysiol.

[19] Beam W (2009). An efficient and accurate new method for locating the F3 position for prefrontal TMS applications. Brain Stimulation.

[20] Sun Y (2016). Indicators for Remission of Suicidal Ideation Following Magnetic Seizure Therapy in Patients With Treatment-Resistant Depression. JAMA Psychiatry.

[21] Sun Y (2018). Magnetic seizure therapy reduces suicidal ideation and produces neuroplasticity in treatment-resistant depression. Transl Psychiatry.

[22] Tononi G, Sporns O, Edelman GM (1994). A measure for brain complexity: relating functional segregation and integration in the nervous system. Proceed Natl Acad Sci US Am.

[23] Costa, M., Goldberger A.L., and Peng C-K., Multiscale entropy analysis of biological signals. Physical review. E, Statistical, nonlinear, and soft matter physics, 2005. 71(1539-3755): p. 021906. 10.1103/PhysRevE.71.021906 15783351

[24] Farzan F (2017). Brain temporal complexity in explaining the therapeutic and cognitive effects of seizure therapy. Brain.

[25] Zewdie E (2020). Safety and tolerability of transcranial magnetic and direct current stimulation in children: Prospective single center evidence from 3.5 million stimulations. Brain Stimulation.

[26] Allen CH, Kluger BM, Buard I (2017). Safety of Transcranial Magnetic Stimulation in Children: A Systematic Review of the Literature. Pediatr Neurol.

[27] Kellner CH (2005). Relief of expressed suicidal intent by ECT: a consortium for research in ECT study. Am J Psychiatr.

[28] Prudic, J. and Sackeim H.A., Electroconvulsive therapy and suicide risk. J Clin Psychiatry, 1999. 60 Suppl 2: p. 104-110; discussion 111-6. 10073397

[29] Daskalakis ZJ (2020). Magnetic seizure therapy (MST) for major depressive disorder. Neuropsychopharmacol.

[30] Kallioniemi E (2019). Magnetic seizure therapy: Towards personalized seizure therapy for major depression. Personalized Med Psychiatr.

[31] Luscher B, Shen Q, Sahir N (2011). The GABAergic deficit hypothesis of major depressive disorder. Mol Psychiatr.

[32] Brambilla P (2003). MRI investigation of temporal lobe structures in bipolar patients. J Psychiatr Res.

[33] Merali Z (2004). Dysregulation in the suicide brain: mRNA expression of corticotropin-releasing hormone receptors and GABA(A) receptor subunits in frontal cortical brain region. J Neurosci.

[34] Poulter MO, et al. GABA(A) Receptor Promoter Hypermethylation in Suicide Brain: Implications for the Involvement of Epigenetic Processes. Biol Psychiatry. 2008. 10.1016/j.biopsych.2008.05.028 18639864

[35] Sequeira A (2007). Patterns of gene expression in the limbic system of suicides with and without major depression. Mol Psychiatry.

[36] Daskalakis ZJ (2002). The mechanisms of interhemispheric inhibition in the human motor cortex. J Physiol.

[37] Voineskos, D., et al., Altered Transcranial Magnetic Stimulation–Electroencephalographic Markers of Inhibition and Excitation in the Dorsolateral Prefrontal Cortex in Major Depressive Disorder. Biological Psychiatry, 2018. 10.1016/j.biopsych.2018.09.032 30503506

[38] Hadas I, et al. Association of Repetitive Transcranial Magnetic Stimulation Treatment With Subgenual Cingulate Hyperactivity in Patients With Major Depressive Disorder: A Secondary Analysis of a Randomized Clinical Trial. JAMA Network Open. 2019;2(6):e195578–8. 10.1001/jamanetworkopen.2019.5578 PMC6551850 31167023

[39] Hadas I (2020). Subgenual cingulate connectivity and hippocampal activation are related to MST therapeutic and adverse effects. Translational Psychiatry.

